# Emotion dynamics and tinnitus: Daily life data from the “TrackYourTinnitus” application

**DOI:** 10.1038/srep31166

**Published:** 2016-08-04

**Authors:** Thomas Probst, Rüdiger Pryss, Berthold Langguth, Winfried Schlee

**Affiliations:** 1 Department of Psychology, Regensburg University, Germany; 2 Department of Psychology and Psychotherapy, Witten/Herdecke University, Germany; 3 Institute of Databases and Information System, Ulm University, Germany; 4 Department of Psychiatry and Psychotherapy, Regensburg University, Germany

## Abstract

It is well established that emotions influence tinnitus, but the role of emotion dynamics remains unclear. The present study investigated emotion dynamics in N = 306 users of the “TrackYourTinnitus” application who completed the Mini-Tinnitus Questionnaire (Mini-TQ) at one assessment point and provided complete data on at least five assessment points for the following state variables: tinnitus loudness, tinnitus distress, arousal, valence. The repeated arousal and valence ratings were used for two operationalizations of emotion dynamics: intra-individual variability of affect intensity (*pulse*) as well as intra-individual variability of affect quality (*spin*). Pearson correlation coefficients showed that the Mini-TQ was positively correlated with pulse (r = 0.19; p < 0.05) as well as with spin (r = 0.12; p < 0.05). Multilevel models revealed the following results: increases in tinnitus loudness were more strongly associated with increases in tinnitus distress at higher levels of pulse as well as at higher levels of spin (both p < 0.05), whereby increases in tinnitus loudness correlated even stronger with increases in tinnitus distress when both pulse as well as spin were high (p < 0.05). Moreover, increases in spin were associated with a less favorable time course of tinnitus loudness (p < 0.05). To conclude, equilibrating emotion dynamics might be a potential target in the prevention and treatment of tinnitus.

Tinnitus, the perception of sound in the absence of a corresponding physical sound source, has prevalence rates ranging from 2 to 26%^1^,^2^,^3^,^4^ and the prevalence rates appear to increase over time^5^. The social and economic costs of tinnitus are enormous. In the Netherlands, for example, the societal costs were estimated to amount to €6.8 billion with €1.9 billion related to health care costs^6^. Costs were found to be even higher in tinnitus patients with depression^6^. Depression is frequent among tinnitus patients^7^ and is stronger associated with tinnitus distress than with tinnitus loudness^8^. Loudness and distress are two components of tinnitus processed in different but interconnected brain networks^9^,^10^. The results that different variables (e. g., depression, brain networks) are differently associated with tinnitus loudness and tinnitus distress fit to the moderate statistical correlations between these two tinnitus components (r = 0.45–0.52) found in large surveys^8^,^11^. A deeper understanding of the psychological processes that put patients at-risk of being distressed by the sensation of tinnitus loudness is of utmost importance as it may provide a framework for the development of specific therapeutic interventions^12^. Moderation and mediation analyses are promising statistical methods to identify psychological and psychopathological mechanisms underlying the loudness-distress relationship. Weise and colleagues analyzed whether depressed mood, anxiety, and tinnitus acceptance mediate the relationship between tinnitus loudness and tinnitus distress^13^. They found that anxiety and tinnitus acceptance explain a significant proportion of the effect tinnitus loudness exerts on tinnitus distress. Depressed mood, however, had no significant mediating effect in their study. This is in contrast to the result that “not feeling low/depressed” was strongly associated with low distress despite high loudness in a large survey^11^. A recent study used ecological momentary assessments of the “TrackYourTinnitus” smartphone application and found that the emotional state “stress” and the two components of emotional states–“valence” and “arousal”–mediate the loudness-distress relationship^14^. Variations of emotional states were also shown to be associated with tinnitus onset and cochlear sensitivity in another study^15^. Although this highlights the role of emotional states in tinnitus, it remains unclear how an individual’s tinnitus is related to her/his emotion dynamics (i. e., variability of emotional states over time^16^,^17^). Despite the high relevance of emotion dynamics (see for example the special section in *Emotion Review*^17^), psychopathology research and behavioral science in general have for a long time neglected dynamic emotional processes because traditional assessment approaches are not well suited to capture dynamic processes^18^. Newer technologies such as smartphone-based applications that allow ambulatory assessments are promising methods to overcome these historical limitations^18^. One example is the “TrackYourTinnitus” (TYT) smartphone application that was used in the study at hand in ecologically valid environments to explore interactions between two dynamic intra-individual processes, emotion dynamics^16^,^17^ and tinnitus^19^,^20^,^21^. The following three research questions were addressed with the TYT daily life data. First, whether emotion dynamics are associated with tinnitus-related psychological problems. Second, whether emotion dynamics moderate the effect current tinnitus loudness exerts on current tinnitus distress. The third research question addressed whether emotion dynamics affect the time course of tinnitus. Identifying factors that contribute to a more positive or more negative prognosis of tinnitus is important to develop preventive and therapeutic strategies. Although previous studies already explored psychological factors influencing the time course of tinnitus^22^,^23^,^24^, these studies focused on treatment-seeking patients, did not use ecological momentary assessments, and did not take emotion dynamics into consideration. In the current study, we had the following three directed hypotheses, since previous research found positive associations between emotion dynamics and psychopathology^16^.

1. Emotion dynamics are positively correlated with tinnitus-related psychological problems.
2. Emotion dynamics moderate the effect of tinnitus loudness on tinnitus distress in a way that tinnitus loudness affects tinnitus distress more strongly in tinnitus sufferers with more emotion dynamics.
3. Increased emotion dynamics are associated with a less favorable time course of tinnitus.

## Methods

The material and the methods were approved by the Ethics Committee of the University Clinic of Regensburg and were carried out in accordance with the approved guidelines. Information that the TYT data will be used for scientific analyses is included in the mobile applications of the “TrackYourTinnitus” as well as on the “TrackYourTinnitus” website and, therefore, the TYT users were informed that the data will be used for scientific purposes. Written consent, however, was not possible to obtain given the nature of the study.

### Material

#### Self-Assessment Manikin (SAM)

The SAM^25^ assesses three components shown to be relevant for the measurement of emotions: 1) arousal, 2) valence, and 3) dominance. In this study, only the arousal and valence components were included to operationalize emotion dynamics (see below). Arousal and valence are considered the two components of emotional states in the circumplex of affect^26^. The component arousal describes the emotional state on a continuum between calm and excitement, whereas the component valence describes the emotional state on an evaluative continuum varying from positive to negative. In the SAM, higher arousal ratings reflect a more excited emotional state, whereas higher valence ratings indicate a more positive emotional state. In the current study, the TYT users completed the arousal and valence ratings repeatedly at different time points during their daily life.

#### Mini-Tinnitus Questionnaire (Mini-TQ)

The Mini-TQ^27^ is a psychometrically sound self-report questionnaire comprising 12 items to rapidly measure tinnitus-related psychological distress. The items are rated on a 3-point Likert-scale and the sum of these 12 items represents the global scale. Higher values indicate more tinnitus-related psychological distress on the items as well as on the global scale. The Mini-TQ is a valid instrument that correlates strongly with the full TQ (r > 0.90) and has a high test-retest reliability (r = 0.89). The use of the following cut-off scores was recommended by Hiller and Goebel^27^: 0–7 = no clinically relevant distress due to the tinnitus; 8–12 = moderately distressed; 13–18 = severely distressed; 19–24 = most severely distressed. In the present study, the Mini-TQ was assessed once per user during the TYT registration process and only those TYT users who completed all 12 items were analyzed.

#### “TrackYourTinnitus” platform

The TYT platform (www.trackyourtinnitus.org)^28^ comprises a website for registration, two mobile applications (for iOS and Android), and a SQL database for the central storage of the collected data. During the registration process, the users are asked to fill out several questionnaires (e. g., the Mini-TQ). After the registration process is finished, the users receive notifications in their daily life to provide “state assessments” that relate to the current moment (see below). In the “standard settings”, these notifications appear on a random basis between 8 a.m. and 10 p.m. In the “custom” settings, however, the users can define their individual schedule. Every time a notification appears, the users were asked to give the following “state assessments”: 1) conscious perception of tinnitus at this moment (subjective rating: yes/no), 2) current tinnitus loudness (subjective rating of current tinnitus loudness on a visual analogue scale, VAS, including a zero value for moments without loudness), 3) current tinnitus distress (subjective rating of current tinnitus distress on a VAS including a zero value for moments without distress), 4) current emotional valence (SAM), 5) current emotional arousal (SAM), 6) current stress level (subjective rating of current stress level on a VAS), 7) current level of concentration (subjective rating of current concentration on a VAS), and 8) an individualized question reflecting the current status of the symptom the user rated to be her/his worst during the registration process. While giving these “state assessments”, the background sound level is measured using the built-in microphone of the smartphone (users could disable this function in the settings). Of all the variables measured by the TYT, only tinnitus loudness, tinnitus distress, valence, arousal, and the Mini-TQ were statistically analyzed in the present study. The data set used for the current study was exported in February 2016.

### Operationalization of emotion dynamics

**Figure 1:**
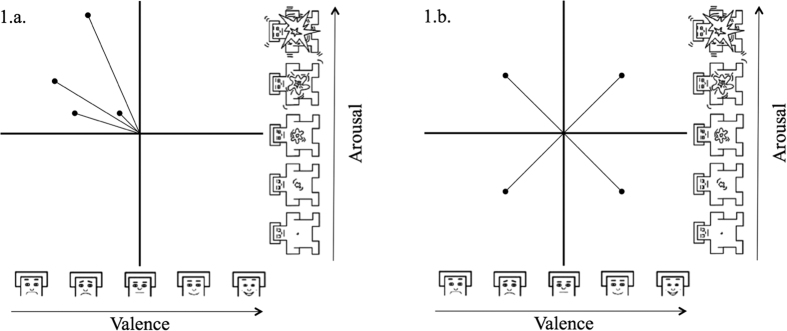
1.a. pulse (affect intensity variability) and 1.b. spin (affect quality variability) according to Kuppens and colleagues^30^. Valence and arousal manikins modified from M. M. Bradley, & P. J. Lang, Measuring emotion: the self-assessment manikin and the semantic differential, Journal of Behavior Therapy and Experimental Psychiatry, 25, 49–59, Elsevier, 1994^25^.

Various operationalizations of emotion dynamics were suggested in the research literature^16^,^29^. The study at hand used a relatively new approach to define emotion dynamics that was introduced by Kuppens and colleagues^30^. Building on the work of Moskowitz, and Zuroff 31, they proposed two intra-individual measures of emotion dynamics depending on how an individual moves within the circumplex of affect that is defined by the dimensions arousal and valence^26^. Using repeated arousal and valence ratings of one individual, “variability of affect intensity” (*pulse*, see Fig. 1) as well as “variability of affect quality” (*spin*, see Fig. 1) can be calculated for this given individual^30^. While pulse defines the degree of variation between experiencing more or less intensive affect, spin reflects the degree of variation between different directions in the affective circumplex. As such, spin operationalizes the extent of qualitatively different feelings regardless of their intensity, whereas pulse stands for the extent how feelings differ in their intensity regardless of the quality of the affective circumplex (i.e., higher or lower valence, higher or lower arousal).

A detailed description for the statistical calculation of pulse and spin can be found in the relevant literature^30^,^32^.

### Statistical analyses

The statistical analyses were performed with SPSS 23.

Means (M), standard deviations (SD), frequencies (n), and percentages (%) were calculated for the sample description.

Pearson’s correlation coefficients were calculated between the Mini-TQ and the intra-individual pulse as well as between the Mini-TQ and the intra-individual spin. Pulse and spin were correlated with the Mini-TQ global scale as well as with each of the 12 Mini-TQ items to investigate our first research question (i. e., whether emotion dynamics are associated with tinnitus-related psychological problems).

To address the second research question (i. e., whether emotion dynamics moderate the effect of tinnitus loudness on tinnitus distress), a multilevel model was performed. The multilevel model included two levels: “state assessments” as level-1 nested within TYT users as level-2. Current tinnitus distress was the dependent variable and current tinnitus loudness was entered as time-varying level-1 covariate. The z-standardized pulse and the z-standardized spin were added as time-invariant level-2 covariates. Regarding the fixed effects, all main and interaction effects were analyzed. The full maximum-likelihood method was used and the intercept was allowed to vary randomly (random intercept model). A moderation model was applied instead of a mediation model for this research question, because, in a mediation model, current tinnitus loudness would function as the antecedent of the potential mediator emotion dynamics which in turn would be expected to influence the outcome variable current tinnitus distress. This appeared inappropriate for the current analysis, since emotion dynamics were operationalized on the user-level (level-2) in the study at hand (the repeated arousal and valence assessments of a TYT user were used to calculate one pulse value and one spin value per user; see “operationalization of emotion dynamics”), whereas current tinnitus loudness as well as current tinnitus distress were measured repeatedly on the assessment-level (level-1), which was nested within the user-level.

For the third research question (i. e., whether emotion dynamics are relevant for the time course of tinnitus), two multilevel models were performed. The multilevel models again included two levels: “state assessments” as level-1 nested within TYT users as level-2. One of these multilevel models evaluated the effect of emotion dynamics on the time course of current tinnitus distress. Therefore, current tinnitus distress was entered as dependent variable, the measurement point of the “state assessment” (set equal to 0 at the first assessment: 0, 1, 2, …) was added as predictor (time course; “slope”) and the z-standardized pulse as well as the z-standardized spin were entered as time-invariant level-2 covariates. The other multilevel model explored the effect of emotion dynamics on the time course of current tinnitus loudness. Hence, current tinnitus loudness was the dependent variable, the measurement point of the “state assessment” (set equal to 0 at the first assessment: 0, 1, 2, …) was the predictor (time course; “slope”), and the z-standardized pulse as well as the z-standardized spin were level-2 covariates. Both multilevel models were estimated with the full maximum-likelihood method and the intercept as well as the slope were allowed to vary randomly (random intercept random slope models). For the random effects, an unstructured variance-covariance matrix was selected. For the fixed effects, all main and interaction effects were investigated.

All statistical analyses were performed two-tailed, no statistical corrections for multiple tests were applied, and the significance value was set to p ≤ 0.05.

### Participants

To investigate our research questions, the following three exclusion criteria were applied to select the sample for the statistical analyses: 1) assessments with a missing value on any of the statistically analyzed variables were excluded. Hence, all users not completing all 12 items of the Mini-TQ were excluded as well as all users not providing a “state assessment” with complete data on tinnitus loudness, tinnitus distress, valence, and arousal. This criterion was applied to have the same sample for the research questions. 2) Every “state assessment” given within a 15 minutes time interval following the previous “state assessment” was excluded. This criterion ensured that there are at least 15 minutes between two assessments as intended in the TYT^28^. 3) Users with less than five “state assessments” were excluded. We determined for this study that at least five observations of the state variables are necessary within a user to meaningfully operationalize her/his intra-individual emotion dynamics (i. e., pulse and spin). It should be kept in mind, however, that the effect of pulse and spin on tinnitus could be more or less strong when using a sample that would result from a different minimum of “state assessments” (e. g., four or six).

**Figure 2:**
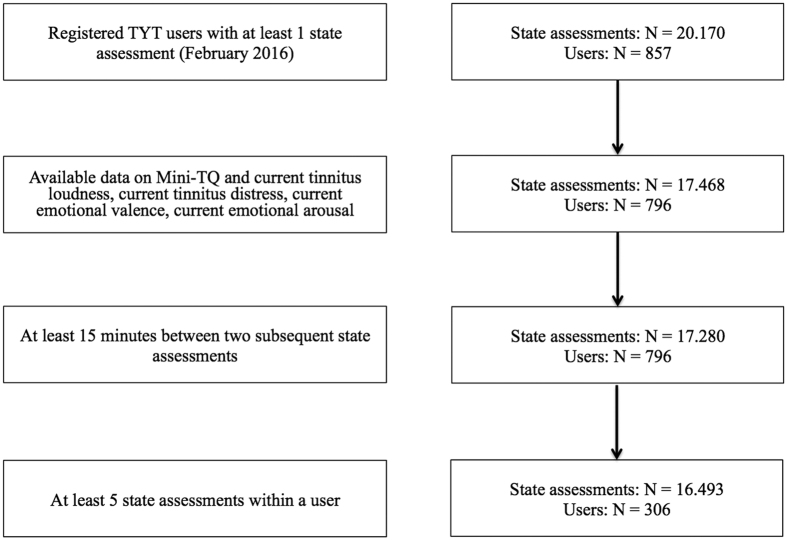
Flow-chart illustrating the amount of excluded “state assessments” and TYT users. TYT = “Track Your Tinnitus” application; Mini-TQ = Mini-Tinnitus Questionnaire.

After the criteria 1–3) were applied, N = 306 tinnitus sufferers of the TYT platform remained available for the statistical analyses. They provided 16.493 “state assessments”. Figure 2 illustrates how many assessments and how many TYT users had to be excluded due to each of the above described exclusion criteria. It can be seen that most of the users had to be excluded because they provided less than five “state assessments” (i. e., due to criterion 3).

The sample description of the TYT users included in this study is shown in Table 1.

## Results

The N = 306 TYT users included in this study provided on average M = 54 “state assessments” (SD = 99). The mean time interval between the users’ first and last assessment point amounted to M = 2 months (SD = 3; range: min = 8 hours; max = 19 months). Across all “state assessments”, the average time interval between one “state assessment” and the subsequent “state assessment” amounted to M = 29 hours (SD = 230; range: min = 15 min; max = 9930 hours). Regarding emotion dynamics, the average pulse was M = 0.12 (SD = 0.04) and the average spin amounted to M = 0.80 (SD = 0.48).

Table 2 shows the results for research question 1, the correlations between tinnitus-related psychological problems measured with the Mini-TQ and intra-individual pulse as well as intra-individual spin. The correlational analyses revealed very low to low but statistically significant positive correlations between tinnitus-related psychological problems and pulse (r = 0.19; p < 0.05) and spin (r = 0.12; p < 0.05), respectively. On the item-level of the Mini-TQ, more significant correlations emerged for pulse than for spin (8 vs. 2).

The fixed effects of the multilevel model addressing research question 2 are displayed in Table 3 (the results of the random effects are in the supplementary material). It can be seen that pulse as well as spin both moderated the effect of tinnitus loudness on tinnitus distress independently from each other: increases in tinnitus loudness correlated significantly stronger with increases in tinnitus distress at higher levels of pulse when statistically holding spin constant (γ = 0.0356; p < 0.01) as well as at higher levels of spin when statistically controlling for pulse (γ = 0.0181; p = 0.01). Compared to these independent effects, increases in tinnitus loudness were even more strongly associated with increases in tinnitus distress when both, pulse and spin, increased (γ = 0.0281; p < 0.01).

The fixed effects of the two multilevel models that were performed in the context of research question 3 are presented in Table 4 (see the supplementary material for the results of the random effects). Table 4 shows that neither pulse nor spin had a significant effect on the time course of tinnitus distress. The time course of tinnitus loudness, however, was significantly influenced by spin (γ = 0.0008; p < 0.01). That means, the higher the affect quality variability, the more the tinnitus loudness increased over time.

## Discussion

This study investigated associations between emotion dynamics and tinnitus in the daily life of tinnitus sufferers using the “TrackYourTinnitus” (TYT) smartphone application. The TYT users received random notifications in their daily life to fill out questionnaires on current tinnitus and current emotions via their mobile phone. Although the attention is directed towards the tinnitus by such notifications, Henry and colleagues could show that the tinnitus does not deteriorate when tinnitus patients receive notifications to assess tinnitus-related and emotional variables in daily life^19^. In the study at hand, emotion dynamics were operationalized by two concepts introduced by Kuppens and co-workers: intra-individual pulse and intra-individual spin. While pulse reflects variability of affect intensity, spin stands for variability of affect quality^30^.

We found that pulse as well as spin are positively correlated with tinnitus-related psychological problems (Mini-TQ) and that pulse as well as spin moderate the effect of tinnitus loudness on tinnitus distress: increases in tinnitus loudness were more strongly associated with increases in tinnitus distress at higher levels of pulse as well as at higher levels of spin, whereby higher pulse in combination with higher spin was most detrimental regarding the loudness-distress relationship. In other words: patients who experience both affect intensity variability and affect quality variability are more severely distressed when tinnitus loudness increases than patients showing either variability of affect intensity or variability of affect quality, whereas patients without emotion dynamics are those with the lowest distress levels related to tinnitus loudness. Another result indicates that higher spin was associated with a less favorable time course of tinnitus loudness, i. e., tinnitus loudness increased over time in patients experiencing more qualitatively different emotions (e. g., sadness, anger, happiness, relaxation). Our results fit to the finding of a recent meta-analysis that emotion dynamics are negatively correlated with psychological well-being and positively associated with psychopathology^16^. While modest correlations between emotion dynamics and psychological well-being were found in the meta-analysis^16^, only very low to low correlations between emotion dynamics and tinnitus-related psychological distress emerged in the present study. The lower correlations could be at least partially attributed to the fact that our tinnitus sample might be quite healthy with regard to psychopathologies because we recruited via a smartphone application and, thus, the sample consisted not only of treatment seeking patients. Adding screenings for depression, anxiety, and other mental health problems to the TYT would allow a more detailed investigation of the psychopathological characteristics. However, administering too many questionnaires could limit the usability of the app in daily life. Therefore, at the time of the current study, only the Mini-TQ is integrated in the TYT as a measure of tinnitus-related psychological problems and only 14% of the investigated app users reported no clinically relevant psychological distress (see Table 1). This indicates that psychological problems are common even among tinnitus sufferers recruited via a smartphone app.

Because no study has yet investigated associations between emotion dynamics and tinnitus distress, our results can only be embedded in research on correlations between emotion dynamics and other psychopathologies. We focus thereby on depression and neuroticism, since these dimensions were shown to be relevant in tinnitus^3^,^7^,^33^,^34^ and were analyzed in previous studies with students (n = 58/127^30^; n = 63^35^) that also used pulse and spin as measures of emotion dynamics. These previous studies could have selected university students to obtain a population of different personality traits and psychological adjustment abilities. One of these studies found a significant medium correlation between spin and depression (r = 0.49) but no significant association between pulse and depression emerged (r = −0.17)^30^. Both studies reported correlations between spin and neuroticism (r = 0.20 to 0.37) as well as between pulse and neuroticism (r = −0.13 to r = 0.11)^30^,^35^. While the correlations between pulse and depression as well as neuroticism did not attain statistical significance in the cited studies, pulse was significantly associated with tinnitus-related psychological problems in the study at hand and pulse even correlated with more items of the Mini-TQ (used to measure tinnitus-related psychological problems) than spin. Pulse and spin, therefore, could be differentially relevant depending on the sample (e. g., students vs. tinnitus patients) or the clinical phenomenon (e. g., depression vs. tinnitus distress) under investigation. In this context, the already cited meta-analysis failed to find relevant differences regarding the effect of emotion dynamics on psychological well-being when clinical and non-clinical samples were compared^16^. Furthermore, this meta-analysis revealed only subtle differences between specific kinds of psychopathologies^16^. However, the research question whether pulse and spin are differentially associated with different psychopathologies has not yet been scrutinized and needs to be addressed in future research.

Moreover, it is crucial to determine whether emotion dynamics are causes of psychopathologies or vice versa^16^. The lack of causality can be seen as a limitation of the present study: emotion dynamics could lead to tinnitus loudness/tinnitus distress or tinnitus loudness/tinnitus distress could be causes of emotion dynamics. Although our results do not allow drawing causal inferences, they could implicate that targeting emotion dynamics might be an option in the prevention and treatment of tinnitus. However, it should be kept in mind that emotional inertia (experiencing more self-predictive emotions) should not be the aim of these interventions, because higher emotional inertia has been shown to be associated with lower psychological well-being^16^. When targeting emotion dynamics, one should try to achieve “an adaptive pattern of emotional change characterized by emotions that have less extreme deviations from their mean level (located around 0) and make smaller consecutive jumps from one point to the next, but at the same time are not very self-predictive, as evidenced by less lingering emotions around the same intensity levels and a strong homeostasis toward a baseline level”^16 page 922^. Besides the lack of causality, the fact that our results rely solely on self-ratings can be considered as a further limitation of this study. In addition to self-ratings future research might use affect detection systems that could rely on brain activity, physiology, speech, face and body language^36^ for a broader operationalization of emotion dynamics. Moreover, psychophysiological measurements of tinnitus loudness such as tinnitus-matching tests could be applied for complementary assessments of tinnitus loudness, even if the reliability and validity of these measures of tinnitus loudness is an ongoing matter of debate^37^,^38^. Tinnitus loudness is usually measured quantitatively on a low-high dimension; a recent study, however, provided evidence that the quality of the tinnitus loudness sensation should also be taken into account. Moring and colleagues found that tinnitus sufferers with a specific type of tinnitus loudness sensation (e. g., ringing, buzzing, hissing, or whooshing) show less functional impairment and less avoidant behavior than tinnitus sufferers with a combination of different types of tinnitus loudness sensations^39^. Therefore, emotional variables such as emotional states and emotion dynamics could be differentially relevant in the loudness-distress relationship depending on the type of tinnitus loudness sensation(s). Due to the fact that each user can provide “state assessments” in the TYT whenever he/she wants to, the range of the time interval between two subsequent “state assessments” is relatively large and this lack of a standardized sampling protocol (e. g., event-contingent or time-contingent) can be seen as another shortcoming of our study. Finally, a main shortcoming of the study at hand is that–as with any online data collection–the robustness/accuracy of the data is difficult to verify. Recent technical developments involving sophisticated plausibility checks for testing data quality might be promising in this context.

Despite these limitations, the study has also several strengths: using pulse and spin instead of a single-dimension measure has been considered to capture the essence of emotion dynamics^30^. Moreover, ecological momentary assessments by a smartphone application provide results of high ecological validity. Scientifically sound mobile health apps have recently been discussed as innovative and promising research strategies in clinical science^40^,^41^.

In summary, we provide first evidence that higher emotion dynamics are associated with more tinnitus-related psychological distress in daily life, that emotion dynamics put tinnitus sufferers at risk of being more distressed by the tinnitus loudness, and that qualitative emotion dynamics are correlated with a less favorable time course of tinnitus loudness. The questions whether equilibrating emotion dynamics can enrich the prevention and treatment of tinnitus or whether equilibrating emotion dynamics is a potential change mechanism in the treatment of tinnitus needs to be evaluated in future clinical studies.

## Additional Information

**How to cite this article**: Probst, T. *et al*. Emotion dynamics and tinnitus: Daily life data from the “TrackYourTinnitus” application. *Sci. Rep.* **6**, 31166; doi: 10.1038/srep31166 (2016).

## Supplementary Material

Supplementary Information

## Figures and Tables

**Table 1:** Sample description.

Variable		Statistics
Gender n (%)	Male	218 (71.48)
	Female	87 (28.52)
Variability of tinnitus (subjective rating) n (%)	No	58 (19.02)
	Yes	247 (80.98)
Family history of tinnitus n (%)	No	238 (78.03)
	Yes	67 (21.97)
Onset relation n (%)	Loud blast of sound	42 (13.73)
	Whiplash	8 (2.61)
	Change in hearing	38 (12.42)
	Stress	80 (26.14)
	Head trauma	14 (4.58)
	Other	124 (40.52)
Mini-TQ severity classification n (%)	No clinically relevant distress (0–7)	43 (14.05)
	Modest distress (8–12)	68 (22.22)
	Severe distress (13–18)	125 (40.85)
	Most severe distress (19–24)	70 (22.88)
Age M (SD)		42.57 (17.56)
Years since tinnitus onset M (SD)		10.07 (11.99)
Mini-TQ global scale M (SD)		14.13 (5.62)
Intra-individual variability (standard deviation) of the tinnitus distress assessments M (SD)		0.18 (0.07)
Intra-individual variability (standard deviation) of the tinnitus loudness assessments M (SD)		0.17 (0.07)

Note: M = Mean; SD = Standard Deviation.

**Table 2:** Correlations between emotion dynamics (pulse and spin) and tinnitus-related psychological distress measured with the Mini-Tinnitus-Questionnaire (Mini-TQ).

Mini-TQ	Pulse r	Spin r
Global scale	0.192 (p = 0.001)	0.120 (p = 0.035)
Item 1: I am aware of the noises from the moment I get up to the moment I sleep	0.057 (p = 0.320)	0.044 (p = 0.447)
Item 2: Because of the noises I worry that there is something seriously wrong with my body	0.140 (p = 0.014)	0.065 (p = 0.259)
Item 3: If the noises continue my life will not be worth living	0.080 (p = 0.164)	0.006 (p = 0.919)
Item 4: I am more irritable with my family and friends because of the noises	0.092 (p = 0.109)	0.091 (p = 0.113)
Item 5: I worry that the noises might damage my physical health	0.119 (p = 0.037)	0.112 (p = 0.050)
Item 6: I find it harder to relax because of the noises	0.130 (p = 0.023)	0.118 (p = 0.038)
Item 7: My noises are often so bad that I cannot ignore them	0.117 (p = 0.041)	0.071 (p = 0.212)
Item 8: It takes me longer to get to sleep because of the noises	0.110 (p = 0.054)	0.075 (p = 0.193)
Item 9: I am more liable to feel low because of the noises	0.157 (p = 0.006)	0.053 (p = 0.360)
Item 10: I often think about whether the noises will ever go away	0.134 (p = 0.019)	0.083 (p = 0.149)
Item 11: I am a victim of my noises	0.190 (p = 0.001)	0.108 (p = 0.059)
Item 12: The noises have affected my concentration	0.143 (p = 0.013)	0.099 (p = 0.085)

Note: r = Pearson correlation coefficient.

**Table 3:** Fixed effects of the multilevel model investigating pulse and spin as moderators of the relationship between current tinnitus loudness on current tinnitus distress.

Parameter	Estimate	SE	df	T-statistics	p-value
Intercept	0.0533	0.0069	401.627	7.775	<0.001
Pulse	−0.0060	0.0073	453.506	−0.827	0.409
Spin	−0.0019	0.0073	441.046	−0.261	0.794
Current tinnitus loudness	0.7068	0.0053	16433.669	132.062	<0.001
Pulse * spin	−0.0078	0.0062	505.366	−1.263	0.207
Current tinnitus loudness* pulse	0.0356	0.0061	16011.413	5.821	<0.001
Current tinnitus loudness * spin	0.0181	0.0060	16138.995	3.000	0.003
Current tinnitus loudness * pulse * spin	0.0281	0.0055	15745.714	5.128	<0.001

Note: Intercept = Current tinnitus distress when statistically controlling for pulse, spin and current tinnitus loudness; SE = Standard Error; df = degrees of freedom. Pulse and spin were z-standardized on level-2 for this analysis. See the supplementary material for the random effects.

**Table 4:** Fixed effects of the multilevel models on the effects of pulse and spin on the time course of tinnitus.

Parameter	Estimate	SE	df	T-statistics	p-value
**Tinnitus distress**					
Intercept1	0.3796	0.0116	312.088	32.734	<0.001
Pulse	0.0168	0.0120	316.062	1.404	0.161
Spin	0.0061	0.0120	315.249	0.508	0.612
Pulse * spin	0.0077	0.0099	320.310	0.783	0.434
Slope1	−0.0006	0.0004	51.323	−1.649	0.105
Slope1 * pulse	0.0003	0.0004	51.916	0.858	0.395
Slope1 * spin	0.0005	0.0004	52.175	1.413	0.164
Slope1 * pulse * spin	0.0006	0.0003	54.241	1.655	0.104
**Tinnitus loudness**					
Intercept2	0.4589	0.0119	313.191	38.482	<0.001
Pulse	0.0100	0.0123	317.175	0.808	0.420
Spin	−0.0027	0.0123	316.541	−0.222	0.824
Pulse * spin	0.0063	0.0102	321.234	0.618	0.537
Slope2	−0.0005	0.0003	42.532	−1.821	0.076
Slope2 * pulse	−0.0001	0.0003	43.080	−0.305	0.762
Slope2 * spin	0.0008	0.0003	44.555	2.848	0.007
Slope2 * pulse * spin	0.0005	0.0003	43.944	1.685	0.099

Note: Intercept1 = Current tinnitus distress at the first “state assessment” when statistically controlling for pulse and spin; Intercept2 = Current tinnitus loudness at the first “state assessment” when statistically controlling for pulse and spin; Slope1 = Changes of current tinnitus distress over time when statistically controlling for pulse and spin; Slope2 = Changes of current tinnitus loudness over time when statistically controlling for pulse and spin; SE = Standard Error; df = degrees of freedom. Pulse and spin were z-standardized on level-2 for these analyses. See the supplementary material for the random effects.
