# Supplementary material for: Emotion dynamics and tinnitus: Daily life data from the “TrackYourTinnitus” application
Source: Sci Rep. 2016 Aug 4;6:31166. doi: 10.1038/srep31166 (PMC4973236; doi:10.1038/srep31166)
Supplement: Supplementary Information [file srep31166-s1.doc]

**Authors**

Thomas Probst*^1^, Rüdiger Pryss^2^, Berthold Langguth^3^, Winfried Schlee^3^

1) Department of Psychology and Psychotherapy, Witten / Herdecke University, Germany

2) Institute of Databases and Information System, Ulm University, Germany

3) Department of Psychiatry and Psychotherapy, Regensburg University, Germany

* Thomas.Probst@uni-wh.de

**Title**

Emotion dynamics and tinnitus. Daily life data from the “TrackYourTinnitus” application.

**Supplementary material**

Random effects of the multilevel model investigating pulse and spin as moderators of the relationship between current tinnitus loudness on current tinnitus distress (Table 3).

| **Parameter** | **Estimate** | **Standard Error** | **Wald Z** | **p-value** |
| --- | --- | --- | --- | --- |
|
| Residual Variance | .011 | .000 | 89.962 | < .01 |
| Intercept Variance | .010 | .001 | 11.532 | < .01 |

Random effects of the multilevel model on the effects of pulse and spin on the time course of tinnitus (Table 4). Unstructured variance-covariance matrix.

| **Tinnitus distress** | | | | |
| --- | --- | --- | --- | --- |
| **Parameter** | **Estimate** | **Standard Error** | **Wald Z** | **p-value** |
|
| Residual Variance | .023 | .000 | 88.547 | < .01 |
| Intercept Variance | .033 | .003 | 11.554 | < .01 |
| Intercept – Slope Covariance | - .000 | .000 | - 1.186 | .24 |
| Slope Variance | .000 | .000 | 3.901 | < .01 |
| **Tinnitus loudness** | | | | |
| **Parameter** | **Estimate** | **Standard Error** | **Wald Z** | **p-value** |
| Residual Variance | .026 | .000 | 88.730 | < .01 |
| Intercept Variance | .035 | .003 | 11.612 | < .01 |
| Intercept – Slope Covariance | .000 | .000 | .576 | .57 |
| Slope Variance | .000 | .000 | 3.505 | < .01 |
